# Self-Supervised Contrastive Learning to Predict the Progression of Alzheimer’s Disease with 3D Amyloid-PET

**DOI:** 10.3390/bioengineering10101141

**Published:** 2023-09-28

**Authors:** Min Gu Kwak, Yi Su, Kewei Chen, David Weidman, Teresa Wu, Fleming Lure, Jing Li, for the Alzheimer’s Disease Neuroimaging Initiative

**Affiliations:** 1 H. Milton Stewart School of Industrial and Systems Engineering, Georgia Institute of Technology, Atlanta, GA 30332, USA; mkwak35@gatech.edu; 2 Banner Alzheimer’s Institute, Phoenix, AZ 85006, USA; yi.su@bannerhealth.com (Y.S.); kewei.chen@bannerhealth.com (K.C.); david.weidman@bannerhealth.com (D.W.); 3 School of Computing and Augmented Intelligence, Arizona State University, Tempe, AZ 85281, USA; teresa.wu@asu.edu; 4 MS Technologies Corporation, Rockville, MD 20850, USA; fleming.lure@mstechnologies.com

**Keywords:** Alzheimer’s disease, mild cognitive impairment, amyloid-PET, self-supervised learning, representation learning

## Abstract

Early diagnosis of Alzheimer’s disease (AD) is an important task that facilitates the development of treatment and prevention strategies, and may potentially improve patient outcomes. Neuroimaging has shown great promise, including the amyloid-PET, which measures the accumulation of amyloid plaques in the brain—a hallmark of AD. It is desirable to train end-to-end deep learning models to predict the progression of AD for individuals at early stages based on 3D amyloid-PET. However, commonly used models are trained in a fully supervised learning manner, and they are inevitably biased toward the given label information. To this end, we propose a selfsupervised contrastive learning method to accurately predict the conversion to AD for individuals with mild cognitive impairment (MCI) with 3D amyloid-PET. The proposed method, SMoCo, uses both labeled and unlabeled data to capture general semantic representations underlying the images. As the downstream task is given as classification of converters vs. non-converters, unlike the general self-supervised learning problem that aims to generate task-agnostic representations, SMoCo additionally utilizes the label information in the pre-training. To demonstrate the performance of our method, we conducted experiments on the Alzheimer’s Disease Neuroimaging Initiative (ADNI) dataset. The results confirmed that the proposed method is capable of providing appropriate data representations, resulting in accurate classification. SMoCo showed the best classification performance over the existing methods, with AUROC = 85.17%, accuracy = 81.09%, sensitivity = 77.39%, and specificity = 82.17%. While SSL has demonstrated great success in other application domains of computer vision, this study provided the initial investigation of using a proposed self-supervised contrastive learning model, SMoCo, to effectively predict MCI conversion to AD based on 3D amyloid-PET.

## 1. Introduction

Alzheimer’s disease (AD) is a neurodegenerative disease and the most common form of dementia. AD symptoms initially include a loss of short-term memory ability, and as the symptoms become worse, cognitive decline occurs. It is estimated that there are 6.7 million individuals aged 65 and older affected by AD in the United States alone in 2023 [1]. Despite several decades of unsuccessful drug development, recent times have signaled a glimmer of hope with the full FDA approval of a novel drug, Leqembi [2]. Moreover, another promising medication, donanemab, is under testing, and showing encouraging early results [3]. Notably, these groundbreaking pharmaceutical developments herald a new era in the fight against AD. Yet, their potential to slow disease progression is contingent upon early administration. There is a strong consensus that the most effective treatment regime should target the early stages of the disease before irreversible brain damage has occurred [4]. Thus, the early identification of an individual’s condition is important [5].

Mild cognitive impairment (MCI) is a prodromal phase of AD when individuals show noticeable signs of memory and cognitive decline, but the symptoms are not severe enough to disrupt their daily activities. MCI is a high-risk stage that 10∼15% of individuals progress to AD each year. It is crucial to identify which MCI individuals will convert to AD (also known as converters), which could provide an opportunity for early intervention to try to slow down the progression. This has been formulated as a classification problem (i.e., classifying MCI individuals into converters vs. non-converters) in the AD literature.

Neuroimaging is an important tool for AD-related assessments, and has demonstrated great potential for predicting MCI conversion to AD. Volumetric magnetic resonance imaging (MRI) and positron emission tomography (PET) are two important neuroimaging modalities [6,7,8,9]. MRI can provide information about the structural alteration of the brain [10]. Training end-to-end deep learning models based on 3D neuroimages has focused more on MRI in past research [7,11]. On the other hand, PET can provide information about functional and pathological changes in the brain. A commonly used PET imaging modality is FDG-PET which measures cerebral glucose metabolism. Amyloid-PET is a promising neuroimaging modality for AD diagnosis, as it measures the accumulation of amyloid plaques in the brain—a hallmark of AD. It is of great interest to use amyloid-PET for converter vs. non-converter classification of MCI patients.

There are two limitations of the existing work we want to tackle in this paper: First, the existing studies using amyloid-PET for MCI conversion classification focused on pre-defined features [12] (e.g., regional amyloid measurements). Building a deep learning model that takes the 3D amyloid-PET images as input without feature engineering will greatly complement the existing studies. Second, most existing approaches are supervised learning models which are trained using labeled data only. Labeled samples can be quite limited, especially for training deep learning models with many parameters. Leveraging other available data sources, such as unlabeled data, has great potential to improve model training. This is especially important for training with amyloid-PET, because this imaging modality is not routinely collected for patients, and thus has a much smaller sample size than MRI.

Self-supervised learning (SSL) is a new machine learning paradigm in which a model is trained to learn general representations of input data (e.g., semantic representations of images) with no label information needed. SSL has gained much popularity recently because of its superior capability of learning representations that are broadly transferable to various downstream tasks by fine-tuning, such as image segmentation, object detection, and classification. Using SSL in a pre-training step, the model trained to perform the downstream task can be less biased to the limited labeled data, thus having better generalizability. SSL has resulted in remarkable improvements in various domain applications, including but not limited to natural images [13], histopathology images [14], autonomous driving [15], and medical images [16,17]. Recent studies have especially focused on medical images. The hybrid architecture of UNet and vision transformer, UNETR, was introduced to learn the sequence representations of 3D input for medical image segmentation. It achieved considerable performance gains for multi-organ, brain tumor, and spleen segmentation tasks [18]. Furthermore, UNETR was improved by adopting the Swin Transformer architecture for efficient training. It also introduced several tailored proxy tasks for proper self-supervised learning in the medical domain [19]. However, only a few studies using SSL in radiology related to AD have been conducted [20]. Furthermore, no study has been performed to predict MCI conversion to AD using 3D amyloid-PET, which motivated our work in this paper.

This study proposes a self-supervised contrastive learning framework, Semi Momentum Contrast (SMoCo), to predict MCI conversion to AD. To the best of our knowledge, it is the first study to leverage the SSL approach for predicting MCI conversion based on 3D amyloid-PET images. We hypothesize that using SSL to obtain general representations from a large amount of unlabeled data can help the model achieve better performance. Acquiring fully labeled datasets in AD research is challenging. The diagnostic process, which requires clinicians evaluating a complex array of information, is both resource-intensive and time-consuming. We address this challenge by employing SSL. Our model is built upon Momentum Contrast (MoCo), a representative existing SSL model that learns representations to minimize a contrastive loss in instance discrimination. To improve MoCo when the downstream task is classification, which is the focus of this paper, we propose SMoCo, which aims to learn more suitable representations for the downstream classification. SMoCo not only leverages unlabeled data, but also exploits label information in the pre-training step. To demonstrate the effectiveness of SMoCo, we conducted experiments on the ADNI dataset and compared it with alternative methods.

## 2. Materials and Methods

### 2.1. Data

This retrospective study was conducted on the Alzheimer’s Disease Neuroimaging Initiative (ADNI) dataset. ADNI is one of the largest datasets for AD studies to date, with the primary goal being to test whether serial MRI, PET, other biological markers, and clinical and neuropsychological assessment can be combined to measure the progression of MCI and early AD. ADNI (http://adni.loni.ucla.edu, accessed on 31 October 2022) was launched in 2003 by the NIH, FDA, private pharmaceutical companies, and nonprofit organizations, as a $60,000,000, 5-year public-private partnership. The primary goal of ADNI has been to test whether MRI, PET, other biological markers, and clinical and neuropsychological assessment can be combined to measure the progression of MCI and early AD. Determination of sensitive and specific markers of very early AD progression is intended to aid researchers and clinicians to develop new treatments and monitor their effectiveness, as well as lessen the time and cost of clinical trials. The Principal Investigator of this initiative is Michael W. Weiner, MD, VA Medical Center and University of California-San Francisco. ADNI is the result of efforts of many co-investigators from a broad range of academic institutions and private corporations, and subjects have been recruited from over 50 sites across the US and Canada. For up-to-date information, please see http://www.adni-info.org/, accessed on 31 October 2022.

**Figure 1:**
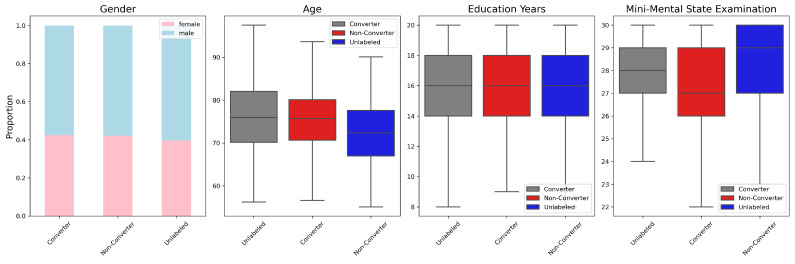
Distributions of demographic and clinical variables in ADNI dataset.

We downloaded 1064 3D amyloid-PET images from 612 individuals. If an individual, with multiple images taken at different times, converts from MCI to AD within 36 months of an image being captured, that image is labeled as converter. Otherwise, it is labeled as non-converter. There were 158 converters and 463 non-converters, and additionally 443 unlabeled MCI images. The unlabeled MCI refers to images that the AD conversion cannot be determined because there are no records at or after 36 months. The demographic and clinical information of the data are demonstrated in Table 1 and Figure 1.

We also downloaded the T1-weighted MR image corresponding to each PET image. The T1-weighted MR images were spatially normalized using the Computational Anatomy Toolbox 12 [21] with Statistical Parametric Mapping [22] and a standard brain atlas from the Montreal Neurological Institute. Then, each PET image was co-registered with the corresponding MRI. The spatially normalized PET images have a size of 121×145×121 and a voxel size of 1.5 mm in depth, height, and width. We applied zero padding and resized the images to a size of 72×72×72 using nearest-neighbor interpolation. The labeled images were split into 80%, 10%, and 10% for training, validation, and testing. All unlabeled images were included in training. The ratio between the labeled and unlabeled data in training is about 1:0.82. After training and validation, the model was applied to the test set to compute performance metrics. The train/validation/test split was repeated 30 times with different random seeds so that the average and standard deviation of test performance metrics could be reported.

### 2.2. Proposed SMoCo

We propose a self-supervised contrastive learning method to predict MCI conversion to AD based on 3D amyloid-PET. It is based on the MoCo, a popular SSL method that has recently set a milestone with its great computational efficiency [23,24]. MoCo aims to capture the semantic representations of images in the pre-training step where no label information is needed. Then, the pre-trained network can be fine-tuned to perform various downstream tasks. To further improve MoCo for classification downstream tasks, we introduce SMoCo. SMoCo refines pre-training representations for classification by leveraging a contrastive loss function that incorporates label information. In the following, we will briefly review MoCo and then introduce SMoCo.

Let $D=D_{L}\cup D_{U}$ be a training dataset, where $D_{L}$ and $D_{U}$ denote the labeled and unlabeled amyloid-PET images, respectively. In the pre-training step, MoCo trains a network by discarding the label information and learning semantic representations of the images through instance discrimination. Formally, given an image $x_{i}\in D$, a stochastic data augmentation t(·) is applied to the same image twice to generate two different views, $x_{i}^{a}=t\left(x_{i}\right)$ and $x_{i}^{+}=t\left(x_{i}\right)$, called the anchor and a positive instance, respectively. By putting $x_{i}^{a}$ and $x_{i}^{+}$ through a query network $f_{\theta }\left(\cdot \right)$ and a key network $f_{\phi }\left(\cdot \right)$ with shared structure, we can obtain their respective representation vectors, $z_{i}^{a}=f_{\theta }\left(x_{i}^{a}\right)$, $z_{i}^{+}=f_{\phi }\left(x_{i}^{+}\right)$, respectively. We should train the network to make $z_{i}^{a}$ and $z_{i}^{+}$ similar, i.e., to “pull” the positive instance toward the anchor because both of them are generated from the same image. In addition, we can draw *K* images other than $x_{i}$ from the training set and apply augmentation t(·) to these images to obtain ${\left\{x_{ik}^{-}\right\}}_{k=1}^{K}$, which are called negative instances. By putting each $x_{ik}^{-}$ through $f_{\phi }\left(\cdot \right)$, we can obtain its representation vector, $z_{ik}^{-}=f_{\phi }\left(x_{ik}^{-}\right)$. We should train the network to make $z_{i}^{a}$ and $z_{ik}^{-}$ dissimilar, i.e., to “push” each negative instance away from the anchor because they are different images. To realize the ideas of the “pull” and “push”, MoCo uses the following loss function:

(1) $$L_{i}^{MoCo}=-\log\frac{\exp(z_{i}^{a}\cdot z_{i}^{+}/\tau )}{\exp\left(z_{i}^{a}\cdot z_{i}^{+}/\tau \right)+\sum_{k=1}^{K}\exp\left(z_{i}^{a}\cdot z_{ik}^{-}/\tau \right)}.$$

τ is a temperature hyperparameter for scaling. Under this loss function, MoCo trains the query network $f_{\theta }\left(\cdot \right)$ and the key network $f_{\phi }\left(\cdot \right)$ with the same structure, but updating parameters ϕ by an exponential moving average of θ. Also, MoCo uses a memory queue to store past representations of negative instances to save computational costs.

Note that MoCo does not use label information, even though the training set D includes a subset of labeled samples $D_{L}$. Leveraging the label information has the potential to learn semantic representations that are more appropriate for the downstream classification task. To achieve this, we propose SMoCo, which “pulls” additional instances toward the anchor. These are instances in the memory queue with the same label as the anchor. Formally, for each labeled image $x_{i}\in D_{L}$, recall that the anchor is obtained by applying augmentation to the image, i.e., $x_{i}^{a}=t\left(x_{i}\right)$. ${\left\{z_{im}^{-}\right\}}_{m=1}^{M_{i}}\subset {\left\{z_{ik}^{-}\right\}}_{k=1}^{K}$ denote a subset of $M_{i}$ instances in the memory queue of length *K* which have the same label as the anchor. To “pull” these instances toward the anchor, we propose the following loss:

(2) $$L_{i}^{Label}=-\frac{1}{\left|M(i;t)\right|}\log\sum_{m\in M_{i}}\frac{\exp(z_{i}^{a}\cdot z_{i}^{+}/\tau )}{\exp\left(z_{i}^{a}\cdot z_{i}^{+}/\tau \right)+\sum_{k=1}^{K}\exp\left(z_{i}^{a}\cdot z_{ik}^{-}/\tau \right)}.$$

**Figure 2:**
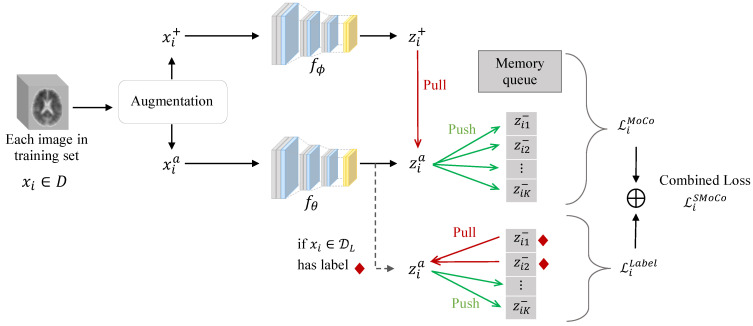
Graphical overview of SMoCo. For a given image $x_{i}$, two augmentations are applied to generate a positive instance $x_{i}^{+}$ and an anchor $x_{i}^{a}$. Both instances are fed into 3D ResNet-50 encoders $f_{\phi }$ and $f_{\theta }$ to obtain representations $z_{i}^{+}$ and $z_{i}^{a}$, respectively. $L_{i}^{MoCo}$ aims to pull $z_{i}^{+}$ toward $z_{i}^{a}$ because they are created from the same image, while pushing other instances in the memory queue away from $z_{i}^{a}$. $L_{i}^{Label}$ leverages label information from the memory queue, ensuring the representations from the same class are pulled closer to $z_{i}^{a}$. $L_{i}^{MoCo}$ and $L_{i}^{Label}$ are combined as the final loss in SMoCo, $L_{i}^{SMoCo}$.

The final SMoCo loss function is defined as a combined loss of (1) and (2):

(3) $$L^{SMoCo}=\sum_{i\in D}L^{MoCo}+\alpha \sum_{i\in D_{L}}L_{i}^{Label},$$

where α is a balancing hyperparameter. It is worth mentioning that, although it may be possible to pull more instances toward the anchor, e.g., by relying on some pseudo-labels of unlabeled images, we chose a more conservative approach in SMoCo based only on labeled images. This is to avoid introducing labeling noise to the learning of semantic representations. Figure 2 provides a graphical overview of SMoCo.

### 2.3. SMoCo Implementation Details and Fine-Tuning

**Figure 3:**
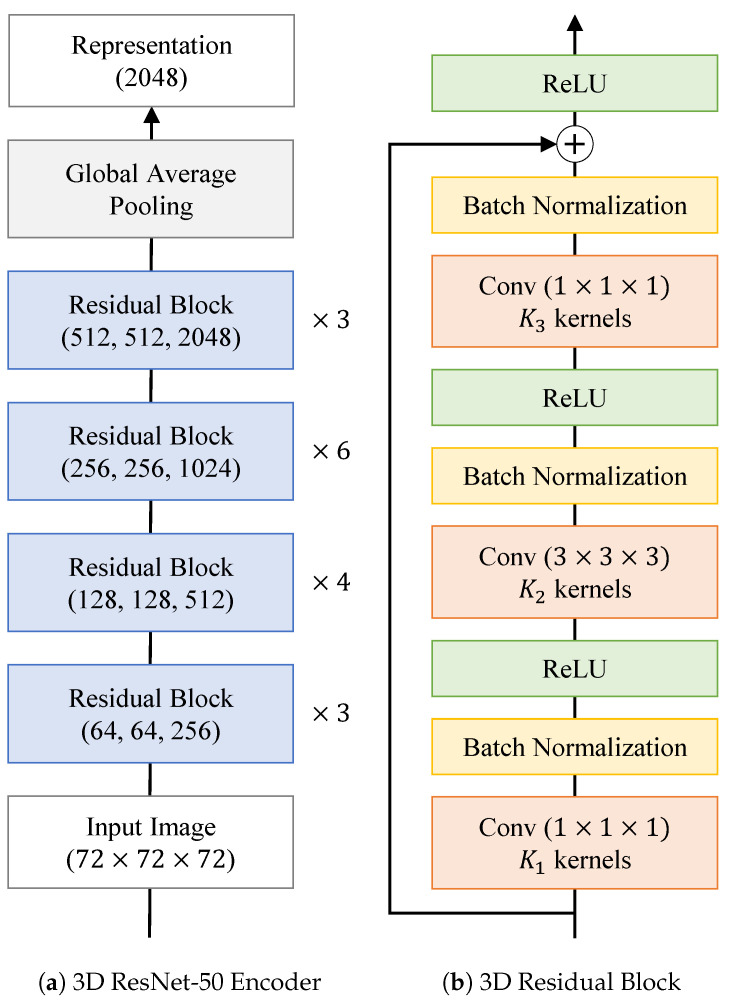
(**a**) Structure of ResNet-50 encoder used for SMoCo (the same encoder is used for $f_{\phi }$ and $f_{\theta }$). The numbers in a bracket denote $K_{1}$, $K_{2}$, and $K_{3}$ of a 3D residual block, respectively. (**b**) Structure of 3D residual block in the encoder.

Inspired by recent works [23,24,25], a 3D ResNet-50 encoder [26] with the fully connected layers replaced by a two-layer multilayer perceptron was chosen for the key and query networks. For the encoder, we replaced the first 7×7×7 convolution layer by a 3×3×3 convolution layer with a stride of one and zero padding of one. The architecture of the 3D ResNet-50 encoder is depicted in Figure 3. The output dimension of the multilayer perceptron was set to 128.

We set τ=0.2,K=1024, and the exponential moving average coefficient as 0.95. Pre-training was performed for 100 epochs with a batch size of 16. The network was optimized using the AdamW [27] optimizer with a momentum of 0.9 and a learning rate of 0.0001. The learning rate is gradually dropped to zero by following a half-cosine schedule. For the proposed SMoCo loss function, values in 0.25,0.5,1,2,3,5 were used to investigate the effect of the balancing hyperparameter α.

After the pre-training is finished, the next step is fine-tuning. Specifically, the multilayer perceptron of the query network was substituted with a single-layer softmax classifier. Then, the network was trained using the pre-trained weights as initial values to minimize the cross-entropy loss for classification based on $D_{L}$. During inference, this fine-tuned network was used to predict the conversion status of each new patient based on their 3D amyloid-PET. All models were implemented with Pytorch [28] and scikit-learn [29].

## 3. Results

### 3.1. Representation Quality Evaluation for Pre-Training Step

**Figure 4:**
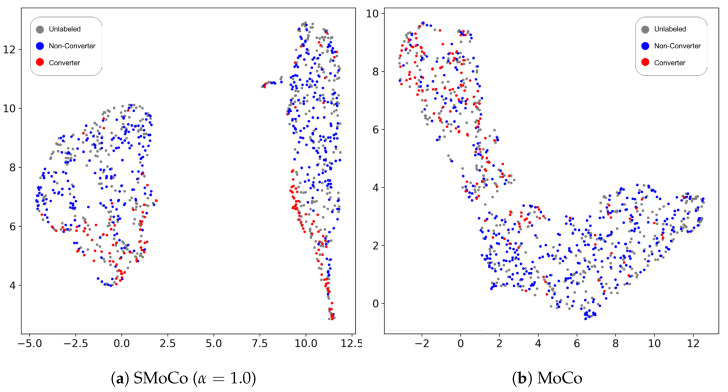
UMAP visualization of the representations of training images. (**a**) SMoCo; (**b**) MoCo. Grey, blue, and red points refer to the unlabeled images, converters, and non-converters, respectively.

In SSL, high-quality representations learned in the pre-training step are important for the downstream task. Here, we compared the representation quality of SMoCo and MoCo. Specifically, we trained SMoCo to minimize the loss in Equation (3) with α=1 and obtained the representation vector for each training sample. The same was performed for MoCo. To visualize the distribution of the training samples, we used Uniform Manifold Approximation and Projection (UMAP) to reduce the dimensionality of the representation vector to two. Figure 4 compares the UMAP representations of MoCo and SMoCo. As shown in Figure 4b, converter and non-converter samples overlapped more when MoCo was used. During learning of the semantic structure of the images, converters and non-converters were separated to some extent because they have different amyloid-PET characteristics. However, the classes were not separated enough, because MoCo is designed to produce general representations, not for the specific classification task. Compared with Figure 4a, we can confirm that SMoCo more clearly separated converters and non-converters.

Furthermore, we compared SMoCo and MoCo using a more quantitative approach than visualization. The idea was that a better representation should entail a better classification of labeled samples based on their representation vectors. To this end, we obtained the representation vectors of samples in the validation set by applying the trained SMoCo and MoCo. Different values of the hyperparameter α were tried for SMoCo. Then, a *k*-nearest neighbor (*k*-NN) algorithm with *k* = 5 was used to classify each validation sample, and the Area under the Receiver Operating Characteristics (AUROC) was reported to appropriately evaluate the models with class imbalance. *k*-NN was adopted because it has been a common choice to evaluate representation quality of SSL [30]. As shown in Table 2, it can be observed that SMoCo enhances the representation quality over MoCo regardless of the value of α. Namely, the proposed loss function helps the model to provide more appropriate representations for classification tasks because, it is designed to perform the given purpose well. The best AUROC is obtained when α=1, which is 4.70%p higher than MoCo. Furthermore, it can be observed that the model performance gradually decreases when α is greater than one. Our interpretation is that the model focused too much on aggregating the instances of the same class so that the instance discrimination task was not properly performed. Overall, a good balance between the two losses combined in SMoCo, i.e., $L^{MoCO}$ and $L^{Label}$, is important to achieve the best representation for differentiating converters vs. non-converters. Based on the results, we fixed α=1 in the remaining experiments.

**Figure 5:**
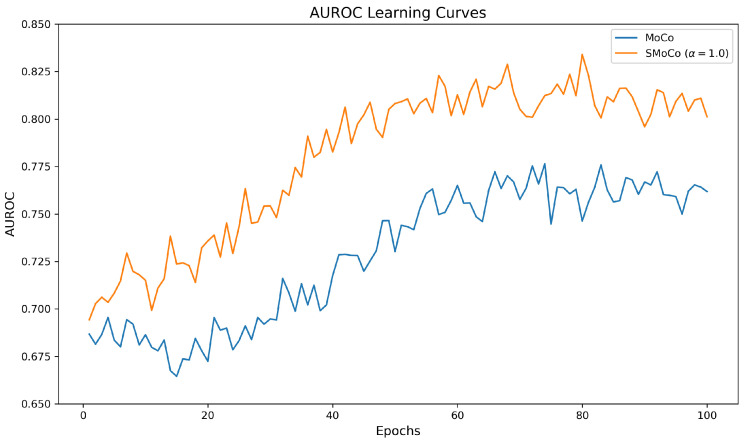
Comparing AUROC of SMoCo and MoCo across training epochs. SMoCo shows faster and efficient training, as well as higher performance than MoCo.

Moreover, we showed the AUROC on validation data along training epochs for both MoCo and SMoCo in Figure 5. Both models demonstrate stable convergence. SMoCo consistently outperforms MoCo with higher AUROC across the training epochs. The SMoCo curve increases earlier than MoCo, indicating that the integration of label information during pre-training can accelerate the model’s ability to capture representations vital for downstream classification.

### 3.2. Classification Performance and Comparison

Finally, we conducted extensive experiments comparing the classification accuracy of our method with a variety of existing methods, including supervised classification, three popular semi-supervised learning methods (pseudo-labeling [31], virtual adversarial training [32], and stochastic weight averaging [33]), and MoCo.

Supervised classification refers to the conventional model that is trained using only labeled data $D_{L}$. It was trained for 100 epochs with an AdamW optimizer using an initial learning rate of 0.0001. The learning rate was also decreased to zero using a half-cosine schedule. The batch size was set to 16. Semi-supervised learning models were trained on $D_{L}$ and $D_{U}$. Unlike SSL, which involves a pre-training and a fine-tuning step, these models were trained at once by incorporating both the cross-entropy loss for $D_{L}$ and an additional loss for $D_{U}$ proposed by the corresponding method. The same training hyperparameters as supervised classification were used.

To evaluate the classification performance of MoCo and SMoCo, we fine-tuned the networks for 10 epochs. Other training hyperparameters were kept the same as supervised classification. These methods are referred to as “MoCo and Fine-Tuning” and “SMoCo and Fine-Tuning” in Table 3. In addition, recognizing that fine-tuning the entire network requires considerable time, another commonly used approach is to use an SSL model as a feature extractor and train a simple classifier based on the representations [34]. We used this approach and trained a random forest classifier [35] with the generated representation vectors from the pre-trained SMoCo. This method is referred to as “SMoCo and Random Forest” in Table 3. Note that random forest cannot be used directly on 3D image data and a prior feature extraction step is needed. Therefore, the “SMoCo and Random Forest” approach is intended to demonstrate the effectiveness of SMoCo as a feature extractor.

Furthermore, noting that our amyloid-PET data has a class imbalance, we applied a resampling technique in every mini-batch to address the problem. We also used an adaptive cutoff strategy to select the threshold for classification probability to assign each sample into a binary class (converters vs. non-converters), which is a recommended strategy under class imbalance [36]. We reported evaluation metrics such as AUROC, accuracy, sensitivity, and specificity.

Table 3 presents the classification results. Overall, classification performance is good in the order of SMoCo, MoCo, semi-supervised methods, and supervised classification. It can be noticed that simply using MoCo enhanced the model performances, especially in terms of AUROC and sensitivity. It improved the prediction of the minority class (converters). We can confirm that the general data representations learned from SSL led to classification performance improvements.

Moreover, the proposed SMoCo further improved the performance of MoCo. Both training a random forest classifier and applying fine-tuning showed better performance than other models. Fine-tuned SMoCo achieved the best performance with considerable gains of 2.16%p of AUROC, 2.72%p of accuracy, 3.27%p of sensitivity, and 3.78%p of specificity than MoCo. This, in turn, proves our original conjecture that “pulling” additional instances with the same label as the labeled images in the training set, as performed by SMoCo, help learn more suitable representations for the downstream classification and bring a substantial performance gap.

## 4. Discussion

Compared to the other application areas of computer vision, the use of SSL in AD studies is quite limited, with only a handful of recent papers focusing on 3D MRI. To classify AD and health controls, an SSL method utilizing a data augmentation technique that mixes medically relevant regions was proposed [37]; a multimodal SSL model was used to combine structural and functional MRI [20]. To predict MCI conversion, a benchmarking study was performed, which revealed that some SSL methods have advantages over supervised pre-training, multitask learning, and multiclass learning [38]. Also, a two-stage model was proposed, which combined transfer learning and self-supervised contrastive learning [11]. However, no study using SSL on 3D amyloid-PET has been found.

On the other hand, there are existing studies using amyloid-PET, by itself or combined with other imaging modalities, for MCI conversion prediction. However, these studies are based on pre-extracted features. One study used the fractal dimension and Shannon entropy as extracted features from amyloid-PET and trained a support vector machine for classification [12]. Another study built a multimodal sparse representation-based classifier based on pre-defined features from various regions of interest obtained from amyloid-PET and MRI [39]. A transfer learning method was proposed to exploit features extracted from regions of interest of amyloid-PET, FDG-PET, and MRI, which can account for missing modalities [40]. In comparison to these studies, our study achieved better and similar performance, but provided an end-to-end method based directly on 3D amyloid-PET without feature engineering.

This study has several limitations. First, SMoCo tends to pull negative instances of the same class as the anchor from the memory queue. This might make the model over-rely on class information, potentially ignoring subtle within-class variations. Recognizing that a class can have diverse patterns, as seen when diagnosing patients with varied symptoms under the same label, a more nuanced approach is required. Like Prototypical Contrastive Learning [41], we can address this by clustering instances and identifying a representative ‘prototype’ for each cluster. This can allow model to account for both the nuances of individual instances and the broader class patterns. Second, this study is based on amyloid-PET data only. Integrating other data modalities such as demographics, clinical records, and MRI has the potential to improve model performance. In a proof-of-concept experiment, we added age, gender, education years, and mini-mental state examination to the fine-tuning stage of SMoCo based on amyloid-PET, as demonstrated in Table 1 and Figure 1. We found improvement over using amyloid-PET alone (AUROC = 86.12%, accuracy = 82.19%, sensitivity = 78.52%, and specificity = 83.71%). We expect further improvement by including MRI, which can be explored in future research. Third, it is well-known that training deep learning models requires large amounts of data, while the sample size of our study is still limited. To expand the training capacity, we could leverage pre-trained 3D networks based on large medical image datasets such as Swin UNETR [42] and Med3D [43]. Last but not least, this study is based on ADNI data. It is important to further validate the proposed method using other datasets than ADNI. To this end, we acquired another public dataset commonly used for AD studies, the Australian Imaging Biomarkers and Lifestyle Study of Ageing (AIBL) [44]. The AIBL dataset contains a limited number of amyloid-PET images with 28 converters and 14 non-converters. To apply our method to the AIBL dataset, we further fine-tuned the previously obtained ADNI-based model using AIBL data. We used 5-fold cross validation, i.e., including 4 folds of AIBL data to further fine-tune the ADNI-based model, testing on the remaining fold, and iterating this process through all folds to compute performance. This resulted in an AUROC of 82.50% on the AIBL dataset, which is comparable to the testing performance using the ADNI dataset (AUROC = 85.17%). This result demonstrated the generalizability of our method, while we acknowledge that the AIBL dataset has a limited sample size. Further validation using larger datasets is needed, and will be explored in future research.

## 5. Conclusions

In summary, our study is among the first ones that leverage SSL to predict MCI conversion to AD based on 3D amyloid-PET Images. Amyloid-PET images have favorable characteristics for early AD diagnosis. We used 3D images to avoid using feature engineering that requires domain knowledge and related tools. The main advantage of SSL is to enable the leveraging of a large amount of unlabeled images to learn general representations, which helps improve the downstream classification task. In AD research, collecting a sufficient amount of diagnosis labels is costly and time-consuming. Therefore, utilizing unlabeled data can be an important benefit. To further strengthen the advantage of SSL, we proposed SMoCo to learn more suitable representations for the downstream classification task of converters and non-converters. Our experimental results showed that SMoCo outperformed a variety of existing SSL, semi-supervised learning, and supervised learning models.

## Figures and Tables

**Table 1:** Demographic and clinical characteristics of the dataset. The ‘Gender’ column represents the proportion of females, while other values denote the mean with standard deviation in parentheses.

	Gender	Age	Education Years	Mini-Mental State Examination
Converter	42.41%	75.34 (7.51)	15.82 (2.81)	26.97 (2.01)
Non-Converter	42.12%	72.45 (7.59)	16.32 (2.73)	28.33 (1.62)
Unlabeled	39.73%	75.79 (8.16)	16.06 (2.63)	27.82 (2.12)

**Table 2:** Representation quality comparison in the pre-training step on validation data. The average values with standard deviations are reported. The best result is **boldfaced**.

Model	MoCo			SMoCo			
α	0	0.25	0.5	1	2	3	5
AUROC	76.37 (3.60)	79.24 (3.32)	80.35 (2.94)	**81.07 (3.02)**	80.63 (3.71)	79.41 (3.40)	78.96 (3.55)

**Table 3:** Classification performance on test data. The average values with standard deviations are reported. The best result is **boldfaced**.

Category	Model	AUROC	Accuracy	Sensitivity	Specificity
Supervised	Supervised Classification	81.53 (3.81)	77.68 (4.01)	73.20 (4.22)	78.89 (3.65)
Semi-Supervised	Pseudo-Labeling	81.89 (3.93)	77.97 (3.53)	73.22 (3.68)	79.18 (3.97)
	Virtual Adversarial Training	82.03 (3.36)	78.13 (3.99)	73.43 (2.98)	78.03 (3.50)
	Stochastic Weight Averaging	82.27 (3.88)	78.19 (3.45)	73.65 (3.39)	78.08 (4.10)
Self-Supervised	MoCo and Fine-Tuning	83.01 (3.59)	78.37 (3.13)	74.23 (2.89)	78.39 (3.77)
	SMoCo and Random Forest	84.86 (3.31)	79.10 (3.09)	74.96 (3.58)	80.03 (3.12)
	SMoCo and Fine-Tuning	**85.17 (2.87)**	**81.09 (3.38)**	**77.39 (2.97)**	**82.17 (3.26)**

## Data Availability

Data used in the preparation of this article were obtained from the Alzheimer’s Disease Neuroimaging Initiative (ADNI) database (adni.loni.usc.edu). As such, the investigators within ADNI contributed to the design and implementation of ADNI and/or provided data but did not participate in the analysis or writing of this report. A complete listing of ADNI investigators can be found at: http://adni.loni.usc.edu/wp-content/uploads/how_to_apply/ADNI_Acknowledgement_List.pdf. The ADNI dataset analyzed in this research work is available at https://adni.loni.usc.edu/ (accessed on 21 August 2021).

## Abbreviations

The following abbreviations are used in this manuscript:

AD	Alzheimer’s disease
ADNI	Alzheimer’s Disease Neuroimaging Initiative
AIBL	Australian Imaging Biomarkers and Lifestyle Study of Ageing
AUROC	Area under the receiver operating characteristic
*k*-NN	*k*-nearest neighbor
MCI	Mild cognitive impairment
MoCo	Momentum contrast
MRI	Magnetic resonance imaging
PET	Positron emission tomography
SSL	Self-supervised learning
UMAP	Uniform manifold approximation and projection
